# Isolation of active peptides from plant hydrolysates that promote Vero cells growth in stirred cultures

**DOI:** 10.1186/1753-6561-5-S1-P116

**Published:** 2011-11-22

**Authors:** Samia Rourou, Rym Hssiki, Héla Kallel

**Affiliations:** 1 Viral Vaccines Research & Development Unit, Institut Pasteur de Tunis, 13, place Pasteur. BP.74 1002 Tunis, Tunisia

## Background

Vero cells are adherent cell lines commonly used for the production of viral vaccines. We had developed an animal component free medium that allows an optimal growth of this cell line in stirred bioreactor [1,2]. We had also showed that Vero cells grown in this medium (called IPT-AFM) sustained rabies virus replication, and resulted in an overall yield comparable to the level obtained in serum-supplemented medium.

IPT-AF medium contains plant hydrolysates, namely soy (Hypep 1510) and wheat gluten hydrolysates (Hypeps 4601 and 4605). These peptones were shown to promote cell attachment and growth. However, although these components are of non-animal origin, their use in vaccine production process has several drawbacks, mainly due variability between lots.

The aim of this work is to identify active peptides from these hydrolysates that show a positive effect on cell adhesion, attachment and growth. For this purpose, the hydrolysates were fractionated using chromatography and precipitation techniques. The effect of the isolated fractions on Vero cells growth were tested in 24 and 6-well cell culture plates using experimental design approach. Fractions that sustain cell growth, were further tested in stirred culture on Cytodex1 microcarriers, to confirm their positive effect on Vero cells growth.

## Materials and methods

***Cell line*:** Vero cells adapted to IPT-AF medium as described in Rourou et al. [2] were used in this study.

***Media*:** M199 medium was purchased from Invitrogen, IPT-AFM was prepared as detailed in Rourou et al. [2]. Hypeps were provided by Sheffield Bio-Science.

### Culture systems

***Static cultures:*** Cells were grown in 24-well plates (Falcon) and 6-well plate experiments « Nunclon Δ». The inoculation density was 2x10^5^ cells/ml and the working volume was equal to 1 ml for the 24-well plate and 3 ml when the cells were grown in 6-well plate. Cells were grown at 37°C in 5% CO_2_ incubator.

***Stirred cultures:*** Cells were grown in 6-well low binding plates (Costar) and in spinner flasks; cultures were inoculated at a cell density of 2x10^5^ cells/ml. The Working volume was equal to 3 ml for the 6-well plate and 200 ml when cultures were performed in spinner flask. Cells were grown on 2 g/l Cytodex 1 at 37°C and 30 rpm.

***Fractionnation protocols*:** Hypeps 1510, 4601 and 4605 were fractionated by either chromatography methods or sequential precipitation with different ethanol concentrations as described in Shen et al. [3].

Sephadex G-25 (GE Healthcare), BioGel P-2 fine (Biorad), Sephadex G-10 (GE Heathcare), HiTrap Q HP (GE Healthcare) and HiTrap SP HP (GE Healthcare) matrixes were tested for the fractionation of the different solutions of Hypeps. Fractionation was monitored by measuring the absorbance of the collected fractions at 214 nm. Collected fractions were desalted, then frozen at -70°C and lyophilized. After lyophilization the fractions were resuspended in M199 so they would be compatible with cell culture.

***Analytical methods:*** Cells grown in static cultures were first detached with the TrypLe Select (Invitrogen) then counted according to the Trypan blue method. Vero cells cultivated on Cytodex 1 microcarriers were counted using the Crystal Violet technique.

***Experimental design and statistical analysis:*** The software Modde 6.0 (Umetrics, Sweden) was used in this study for the design of the experiments and the statistical analysis of the data.

## Results

Sephadex G-10, Sephadex G-25 and Biogel fine-2 were used to fractionate Hypeps 4605 and 4601. However, none of these matrixes was efficient.

Anion and cation exchange chromatography were therefore used as an alternative method; two pH levels were tested: 5 and 8. Although these methods allowed the isolation of different fractions for each peptone, none of them had enhanced Vero cells when the cells were cultivated in 24-well culture plates. In addition, most of these fractions showed a toxic effect on cell growth.

Sequential precipitation with different ethanol concentration was also applied for the fractionation of the three peptones. The fractionation was conducted as described by Shen et al. [3]; 5 fractions were obtained for Hypep 4605 whereas for Hypep 4601 and Hypep 1510, 4 fractions were obtained for each. The effects of the isolated fractions on Vero cells growth were investigated in 24-well plates using a full factorial experimental design; 83 combinations were assessed in duplicate. Two combinations of fractions that show a cell growth comparable to that obtained in IPT-AF medium (positive control), were selected (combinations 1 and 2). The identified combinations were also tested in 6-well plates on 2 g/l Cytodex 1 microcarriers. The highest cell density level reached under these conditions was similar to that achieved in IPT-AF medium.

**Figure 1:**
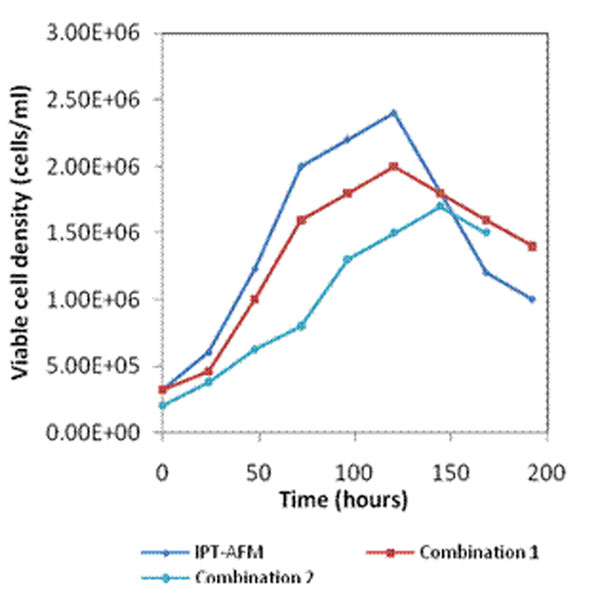
Vero cells on 2 g/l Cytodex 1 in spinner flask, in different media

These combinations were further tested in spinner flask on Cytodex1 microcarriers, to confirm their positive effect on Vero cells growth. Data shown in Figure 1, indicate that cell density level reached 2x10^6^ cells/ml after 5 days of culture when Vero cells were grown in combination 1. Such level was slightly lower than that obtained in IPT-AF medium (2x10^6^ cells/ml versus 2.4x10^6^ cells/ml). However, Vero cell growth in combination 2 was less efficient; the highest cell density obtained in this medium was equal to 1.7x10^6^ cells/ml. Thus, combination 1 appears to be more suitable for Vero cells on Cytodex 1 microcarriers.

## Conclusions

Sephadex G-25, Sephadex G-10, Biogel-fine and ion exchange chromatography matrixes were not efficient for Hypeps fractionation. Sequential precipitation with ethanol appears to be the best method to isolate various fractions that promote Vero cells growth. Two Combinations (1 & 2) were identified as the best in terms of cell density and cell attachment. Spinner cultures demonstrated that these combinations are suitable for Vero cells growth on Cytodex 1. Further fractionation and characterization of these compounds are ongoing to identify the active components.
